# Targeting *BRCA* Deficiency in Breast Cancer: What are the Clinical Evidences and the Next Perspectives?

**DOI:** 10.3390/cancers10120506

**Published:** 2018-12-11

**Authors:** Emanuel Nicolas, François Bertucci, Renaud Sabatier, Anthony Gonçalves

**Affiliations:** 1 Department of Medical Oncology, Institut Paoli-Calmettes, 13009 Marseille, France; nicolase@ipc.unicancer.fr (E.N.); BERTUCCIF@ipc.unicancer.fr (F.B.); SABATIERR@ipc.unicancer.fr (R.S.); 2 CRCM-Predictive Oncology laboratory, Institut Paoli-Calmettes, Inserm U1068, CNRS UMR7258, Aix-Marseille Univ, 13009 Marseille, France

**Keywords:** BRCA, DNA-damaging agents, platinum, poly(ADP)-ribose polymerase

## Abstract

Breast cancers (BC) associated with germline mutations of *BRCA1/2* represent 3–5% of cases. *BRCA1/2*-associated BC have biological features leading to genomic instability and potential sensitivity to DNA damaging agents, including poly(ADP-ribose) polymerase (PARP) and platinum agents. In this review, we will summarize clinical trials of chemotherapy and PARP inhibitors (PARPi), alone or in combination, at the early or late stage of *BRCA1/2*-associated BC. We will also present the mechanisms of resistance to PARPi as well as the new therapeutic strategies of association with PARPi. Finally, we will discuss under which conditions the use of DNA damaging agents can be extended to the *BRCA1/2*-wild type population, the *BRCA*ness concept.

## 1. Introduction

With more than 2 million new cases and nearly 630,000 estimated deaths worldwide in 2018, breast cancer remains the most frequent female cancer as well as the first cause of death by cancer in women [1]. Germline mutations in either the *BRCA1* or *BRCA2 (BRCA1/2)* gene account for approximately 2–3% of all breast cancer, but around 30% of hereditary breast cancer [2,3,4]. These mutations are particularly frequent in the young female population, estimated at 12% in patients under 40, regardless of their family history [5,6]. *BRCA1/2* germline mutations have the highest identifiable life-time risk of developing breast cancer ranging from 56 to 80% [2,7,8,9].

Breast cancers arising from *BRCA1/2* germline mutation carriers are associated with a lack of expression and/or function of the corresponding protein, which induces genomic instability. Indeed, *BRCA1* and *BRCA2* proteins are involved in the repair of DNA damage such as double-strand breaks (DSBs) during S and G2 phases, by intervening in homologous recombination (HR) steps [10,11]. In response to DNA damage, *BRCA2* brings RAD51 to the DSB site and provides the repair [12]. While *BRCA2* is directly involved in RAD51-mediated repair, *BRCA1* appears to act upstream in a more complex mechanism via interaction with other proteins [13,14,15]. HR repair is a “conservative”, potentially error-free, mechanism which restores the original DNA sequence at the site of DNA damage [10,16]. When a cell is deficient in functional HR repair, non-conservative forms of DNA repair such as non-homologous end joining (NHEJ) become dominant [17]. The preferential use of these error-prone DNA repair mechanisms leads to a genomic instability that may favor tumorigenesis [11,16,18]. 

While displaying similar roles in HR, *BRCA1*- and *BRCA2*-associated breast cancer have very different pathology and biology. Thus, more than 80% of *BRCA1*-associated breast cancer correspond to triple-negative breast cancer (TNBCs) [19]. On the other hand, only 10 to 20% of TNBCs carry *BRCA1* mutation [20,21,22]. Histologically, *BRCA1*-associated tumors are frequently associated with a high histological grade, high proliferation indices, invasive borders and lymphocytic infiltrates. They express myoepithelial cell-type cytokeratins (CK 5/6, CK14 and CK17) [19]. This features leads to the idea that tumours arising in *BRCA1* mutation carriers have a basal-like phenotype [23]. In contrast to *BRCA-1* tumours, breast cancer arising from *BRCA2* mutation do not differ from sporadic cancer with regard to distribution of breast cancer subtypes, the luminal forms expressing estrogen and progesterone receptors being the most frequent [24,25]. 

The predominant role of *BRCA1/2* genes in DNA repair process, as well as their functional failure in *BRCA1/2*-associated breast cancer, has been the basis for developing novel therapeutic approaches directly or indirectly targeting this pathway, including specific cytotoxic agents such as platinum compound and poly(ADP-ribose) polymerase (PARP) inhibitors. This review will address recent clinical evidences supporting the role of these treatments, as well as other cytotoxics in *BRCA1/2*-associated breast cancer, and will examine how novel emerging strategies may be built on these promising results.

## 2. *BRCA1/2*-Associated Breast Cancer and Cytotoxic Chemotherapy

### 2.1. Response and Outcome in Advanced Breast Cancer Receiving Chemotherapy According to BRCA Status

In a retrospective study, Kriege et al. analysed 121 patients with *BRCA1/2*-associated metastatic breast cancer (93 *BRCA1* and 28 *BRCA2*) treated with first-line chemotherapy and compared them with those of 121 matched sporadic metastatic breast cancer [26]. Most of the chemotherapy regimen were anthracycline-based, taxane-based or cyclophosphamide-methotrexate-5FU (CMF) association. Platinum agents were underrepresented in this population. Objective response rate was significantly higher in the BRCA2 -related group compared to sporadic cases (89% vs. 50% *p* = 0.001). Progression-free survival (PFS) and overall survival (OS) were also significantly longer in the *BRCA-2* group compared to the sporadic population (multivariate hazard ratio at 0.64, *p* = 0.04 and 0.53, *p* = 0.05, respectively). Yet, no significant response and PFS advantage was demonstrated in the *BRCA1* group. In another retrospective cohort of 195 women with metastatic breast cancer treated at the MD Anderson Cancer Center, including 30 *BRCA1*- and 11 *BRCA2*-associated breast cancer receiving various treatments, *BRCA* non-carriers and *BRCA2* carriers had a longer time-to progression and OS compared to *BRCA1* carriers (median time-to progressio*n* = 1.3 vs. 0.9 vs. 0.7 years, *p* = 0.31, and median OS = 4.88 vs. 4.94 vs. 1.34 years, *p* = 0.0065, respectively). Such an effect was not maintained in a multivariate analysis and TNBCs were associated with poor prognosis, regardless of BRCA status [27].

### 2.2. Pattern of Response to Cytotoxic Chemotherapy in BRCA1/2-Associated Breast Cancer

It has been hypothesized that *BRCA1/2*-associated breast cancers have a specific pattern of response according to chemotherapies and their corresponding mechanisms of action.

#### 2.2.1. Anthracyclines

Anthracyclines can induce DSBs via inhibition of topo-isomerase II. Yet, the impact of *BRCA* status on their clinical efficacy remains discussed. In the advanced setting, anthracycline-based regimen was associated with a non-significant trend toward a lower risk of progression in *BRCA1/2*-associated breast cancer compared to sporadic breast cancer [26]. It should be noted that in this population, some patients (19/147) have benefited from a maintenance chemotherapy regimen based on cyclophosphamide after the anthracycline-based regimen. In the neoadjuvant setting, most of the studies evaluating anthracycline-based regimen reported higher pathological complete response (pCR) rates in the *BRCA1/2* population compared to sporadic forms [28,29]. However, the neoadjuvant chemotherapy regimens often consisted of a combination of anthracyclines (doxorubicin) and alkylating agent (cyclophosphamide). It is therefore difficult to conclude that the pCR is exclusively related to anthracyclines.

#### 2.2.2. Taxanes

Patients carrying *BRCA-1* mutation and treated with anti-microtubule agents such as taxanes may have worse outcomes than the sporadic population. Pre-clinical studies suggest that *BRCA1* defective cells may be resistant to taxanes [30]. *BRCA1* protein is involved in facilitating apoptosis in cells with disrupted mitotic spindle formation. Resistance in the *BRCA1* mutated population may be related to the premature inactivation of the spindle checkpoint [31]. 

In a retrospective monocentric Dutch series, 35 *BRCA1-* and 13 *BRCA2*-associated advanced breast cancers receiving either paclitaxel or docetaxel were compared with 95 sporadic forms. Response rate and PFS were significantly lower in patients carrying *BRCA1* mutations (23% versus 38%, *p* < 0.001 and 2.2 months versus 4.9 months, *p* = 0.04) compared to sporadic breast cancers, most of the difference being driven by hormone receptor-negative tumors [32]. By contrast, patients carrying *BRCA2* mutations (all but one being ER-positive) had a significantly higher response rate than sporadic cancer. 

Of note, the combination of taxanes with anthracyclines in a neoadjuvant setting was associated with better outcomes in the *BRCA1-*associated cancer. Thus, Arun et al reported 46% of pCR in the *BRCA1* mutation carriers while the sporadic breast cancer patients showed 22% [33]. In another retrospective study involving TNBC patients receiving AC-paclitaxel neoadjuvant treatment, 34 *BRCA1* carriers were compared to 43 non-carriers: the pCR rate was higher in *BRCA1* (68% versus 37%, *p* = 0.01) but did not translate into superior survival [34]. More recently, 493 TNBC patients from the GeparQuinto trial (evaluating the addition of bevacizumab to sequential EC- docetaxel), were examined for *BRCA1/2* germline mutations. Irrespective of the treatment arm, the pCR rate was higher in *BRCA1/2* carriers (50%, versus 31.5% in patients without a mutation, *p* = 0.001) and the disease-free survival (DFS) was also better (HR, 0.644; 95% CI, 0.415 to 0.998; *p* = 0.047) [35]. 

#### 2.2.3. Platinum Agents

Platinum compounds interact with DNA to form intrastrand adduct and interstrand cross-links [36]. The lack of functional *BRCA* 1 and 2 proteins causes a defect in the repair of DSBs via the HR and thus may favor a higher sensitivity to these agents [37]. In the neoadjuvant setting, platinum regimens have shown a greater activity in the breast cancer arising from *BRCA*1/2 germline mutation (Table 1) [29,38,39]. In a first retrospective study, Birsky et al. included 6.903 polish patients with breast cancer treated with neoadjuvant chemotherapy, including 102 patients with a germline *BRCA1* mutation [29]. Twelve of these patients were treated with cisplatin and 10 (83%) achieved pCR. These results were confirmed in a larger cohort of 107 Polish stage 1 to 3 patients with a *BRCA1* mutation treated exclusively with neoadjuvant cisplatin (75 mg/m^2^; four cycles) prior to mastectomy [39]. A pCR was observed in 65 of the 107 patients (61%). Comparatively, only 18% of 77 Polish *BRCA1* carriers who received another form of neoadjuvant chemotherapy achieved a pCR. In a small phase II study evaluating neoadjuvant cisplatin in 28 TNBC patients, the two patients with *BRCA1* germline mutation had pCR [40]. However, in an ancillary analysis of TNBC patients enrolled in the GeparSixto trial, which evaluated the addition of carboplatin to an anthracycline/taxane/bevacizumab-based regimen, only *BRCA1/2* wild-type patients seemed to benefit from carboplatin [41].

In the metastatic setting, using platinum agents to treat *BRCA1/2*-associated breast cancer appears to be a reasonable option, even after the first line of treatment (Table 1). In a phase II single-arm trial Birsky et al. included 20 women with *BRCA1*-associated metastatic breast cancer, 55% of whom were at least in second line of treatment [42]. They were treated with cisplatin 75 mg/m^2^ every 3 weeks for six cycles. Overall response rate (ORR) was 80%, including nine patients (45%) with complete response and 7 patients (35%) with partial response. Median PFS and OS were respectively 12 and 30 months. The response rate seemed similar between patients treated in the first-line setting (89%) or beyond (73%), and was higher in ER-negative (eight out of 15) than in ER-positive (one out of five) patients. 

In the same setting, the TBRC009 trial was a phase II study with 86 TNBC metastatic breast cancer including 11 patients with *BRCA1/2* germline mutation [43]. The patient population was treated either with cisplatin 75 mg/m^2^ or carboplatin AUC6 once every 3 weeks. The ORR was 54.5% in patients with *BRCA1/2* mutation but reached only 25.6% (95% CI, 16.8% to 36%) in the overall population; however, there was no difference in survival (PFS and OS) and none of *BRCA1/2* carriers were found in the six patients with very long-term duration of response (as defined as alive, progression-free, and not receiving any therapy at a median of more than 4 years after platinum treatment). Of note, patients treated with cisplatin showed a numerically higher response rate than patients treated with carboplatin (32.2% and 18.7% respectively), like patients treated in the first-line (29%) versus second-line therapy (11.8%). There are only a few randomized data that compared platinum agents to standard of care in metastatic breast cancer patients for whom *BRCA1/2* germline status is known. The *TNT* trial, a randomized phase III study, included metastatic breast cancer patients with TNBC or *BRCA1/2* germline mutation [44]. A total of 376 patients, mostly in the first-line of treatment, including 43 subjects with *BRCA1/2* germline mutation, were randomly assigned to receive carboplatin (AUC 6 every 3 weeks) or docetaxel (100 mg/m^2^ every 3 weeks) for six to eight cycles or until disease progression, with crossover possible on disease progression. In the overall population of the study, there was no superiority of carboplatin over docetaxel. However, in the population with *BRCA1/2* germline mutation, the ORR was twice higher in the patients receiving carboplatin (68% vs. 38%; absolute difference 34.7% (95%CI 6.3 to 61.1) *p* = 0.03). *BRCA1/2* patients treated with carboplatin also experienced significantly higher PFS (6.8 vs. 4.4 months) but there was no difference in OS, which may be due to the cross-over design. In addition, carboplatin appeared to be better tolerated than docetaxel. Thus, this study may support the importance of early detection of *BRCA*1/2 mutation in the population of metastatic TNBC patients, since it may allow selecting a cytotoxic drug with better efficacy in this population.

Of note, platinum agents have only shown a limited activity in unselected advanced breast cancer patients, except in chemotherapy-naive disease [45,46]. However, there may be a rationale for treating TNBC patients with platinum-based chemotherapy even in the absence of documented *BRCA* germline mutation, notably because of the similarities between profiles of *BRCA1-*deficient and TNBCs [47]. These tumors frequently share a basal-like gene expression profile, common *TP53* mutations, and a high burden of genomic aberrations such as loss of heterozygosity [23,48,49].

Thus, it has been hypothesized that genomic instability induced by basal-like cancer may also favor sensitivity to platinum agents [50]. A meta-analysis reporting 717 patients from seven studies confirmed that patients treated with platinum-based chemotherapy who had TNBC showed better outcomes in the neoadjuvant setting than patients with non-TNBC: the clinical complete response (cCR) and the pCR rates were significantly higher in women with TNBC compared to the rest of the population (OR, 2.68; 95% CI, 1.69–6.57; *p* = 0.03 and OR, 2.89; 95% CI, 1.28, 6.53; *p* = 0.01, respectively) [51]. However, in the advanced or metastatic setting, the response rates were not significantly different. In a more recent meta-analysis of nine randomized clinical trials and more than 2100 TNBC patients treated with or without carboplatin, platinum-based neoadjuvant chemotherapy increased the pCR rate from 37.0% to 52.1% (OR 1.96, 95% CI 1.46–2.62, *p * <  0.001), even after restricting the analysis to trials using standard regimen with weekly paclitaxel (with or without carboplatin) followed by anthracycline and cyclophosphamide (OR 2.53, 95% CI 1.37–4.66, *p*  =  0.003). Yet, no impact on event-free survival and OS was identified. In addition, in the 96 patients with *BRCA1/2* germline mutations, there was no detectable impact of carboplatin on pCR (OR 1.17, 95% CI 0.51–2.67, *p*  =  0.711) [52].

#### 2.2.4. Alkylating Agents

Alkylating agents are mechanistically related to platinum compounds, but their specific impact in breast cancer patients with *BRCA1/2* germline mutation remains unclear. Data from a retrospective study indicate that *BRCA1/2* carriers advanced breast cancer patients receiving CMF-like regimen have similar outcomes than wild-type subjects [26]. Yet, in the neoadjuvant setting, *BRCA1* carriers receiving neoadjuvant CMF-regimen had a pCR rate as low as 7%. In a retrospective study evaluating high-dose alkylating agents with autologous hematological stem cell transplantation in 231 advanced breast cancer patients, a small group of 15 *BRCA1/2* carriers had a highly favorable survival outcome, in spite of several adverse prognostic factors, such as younger age, visceral disease, more metastatic sites and more TNBC [53].

More recently, trabectedin, a marine-derived tetrahydroisoquinoline currently produced by chemical synthesis and approved for treating soft tissue sarcoma and relapsed platinum-sensitive ovarian cancer, was evaluated in *BRCA1/2* carriers with advanced breast cancer. Trabectedin binds to the DNA minor groove, forming adducts that prevent effective DNA repair by the transcription coupled nucleotide excision repair machinery and eventually result in cell death; it causes rapid formation of DNA DSBs and loss of HR repair is associated with the persistence of unrepaired DSBs after removal of trabectedin. In a phase II study enrolling 40 heavily pre-treated metastatic breast cancer patients with *BRCA1/2* germline mutations, a response rate of 17% was observed and 40% had clinical benefit [54]. In a subset analysis of the latter study, the response rate was higher in *BRCA2* (33%) than in *BRCA1* (9%) carriers as were the rate of disease stabilization for at least 4 months (25% versus 9%) and median progression-free survival (4.7 versus 2.5 months) [55].

## 3. *BRCA1/2*-Associated Breast Cancer and PARP Inhibitors

### 3.1. PARP Inhibitors and Synthetic Lethality

**Figure 1:**
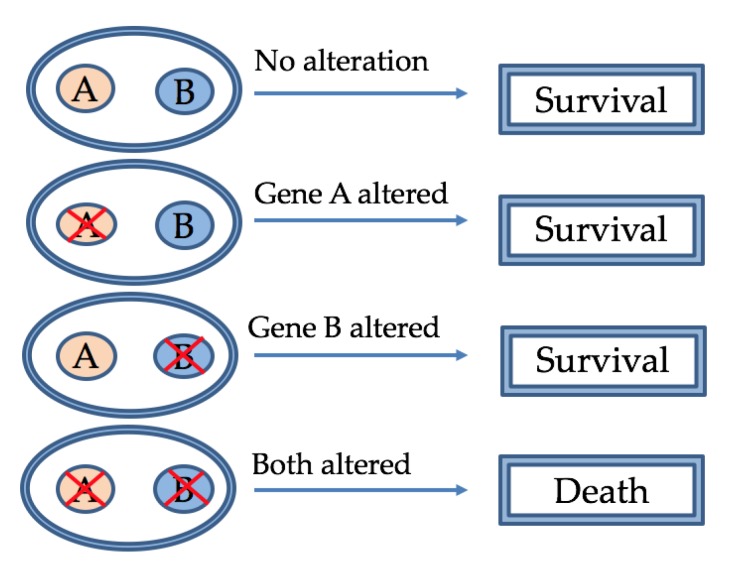
Synthetic lethality: Synthetic lethality occurs when the simultaneous mutation of two genes is lethal for the cell, while mutation in each individual gene is not.

Poly(ADP-ribose) polymerase (PARP) inhibitors belong to a class of drugs that operate on the principle of “synthetic lethality” [56]. “Synthetic lethality” is a concept according to which two genes are synthetic lethal if mutation of either alone is compatible with viability but mutation of both leads to death (Figure 1) [57]. PARPs are a large family of multifunctional enzymes that plays a key role in the repair of DNA single-strand breaks (SSBs) through the repair of base excisions. PARP1 binds damaged DNA at SSBs and other DNA lesions, which causes a series of allosteric changes in the structure of PARP1 that activate its catalytic function [58]. Once activated, PARP-1 poly-ADP ribosylates various target proteins located at the site of DNA damage, including topoisomerases, histones, and PARP-1 itself, all signaling for DNA SSBs and DSBs repair.

Inhibition of PARP leads to accumulation of SSB, which can lead to DSBs at the replication fork and thus to the death of HR-deficient cells such as the mutant *BRCA 1* or *2* mutant. In this way, Farmer et al. [59] and Bryant et al. [60] showed a specific interaction between PARP inhibition and *BRCA1/2* mutation which can lead to synthetic lethality.

New findings suggest that PARP inhibitors (PARPi) may have a predominant cytotoxic mechanism by trapping PARP-DNA complexes on SSB sites [61]. Trapped PARP–DNA complexes are more cytotoxic than unrepaired SSBs and the ability of some PARPi to trap PARP1 may be a predictor of in vitro cytotoxicity in *BRCA*-mutant cells [61,62]. The results of the main clinical trials including PARPi are summarized in Table 2.

### 3.2. Olaparib

In 2009, Fong et al. [63] reported the first phase I trial that evaluated PARPi (olaparib) in metastatic breast cancer. Sixty multi-treated patients including nine with metastatic breast cancer were given olaparib from 10 mg daily for 2 of every 3 weeks to 600 mg twice daily continuously. Three of the metastatic breast cancers carried germinal *BRCA* mutation. Out of these three patients, one had a complete response that remained for more than 60 weeks. Another one had stable disease for 7 months. Olaparib showed an acceptable safety profile and the main adverse events include digestive (such as nausea/vomiting, anorexia and diarrhea) and hematological toxicities (anemia, thrombocytopenia or neutropenia) as well as asthenia.

These results led to three phase II studies, evaluating olaparib in breast cancer [64,65,66]. Tutt et al. assessed 54 women with locally advanced or metastatic breast cancer associated with *BRCA1/2* mutation, divided into two cohorts of 27 patients each [64]. The first cohort (27 patients) was treated with 400 mg twice daily (phase 1 maximum tolerated dose) and the second cohort (27 patients) with 100 mg twice daily (phase 1 lower PARP inhibitory dose). Most patients had already received anthracycline and taxane regimen. Overall response rate was 41% (11 of 27 patients) in the first cohort, including 1 CR and 11 PR, and 22% (six of 27 patients) in the second cohort, including 6 PR. On the contrary, Gelmon et al. [65] reported no objective response for olaparib in a small cohort TNBC patients, but some stable diseases were observed and patients with a *BRCA* germline mutation had PFS twice longer than the overall breast cancer population (3.6 months vs. 1.8 months). Finally, Kaufman et al reported an objective response of only 12.6% in a breast cancer population composed exclusively of heavily pre-treated *BRCA 1/2*-associated tumors [66].

Recently, Robson et al. reported the first randomized, open- label, phase III trial which compared olaparib alone to chemotherapy in patients with HER2-negative metastatic breast cancer carrying *BRCA1/2* germline mutations (*Olympi*AD trial) [67]. They received olaparib (300 mg twice daily) or standard chemotherapy (eribulin, capecitabine, or vinorelbine) selected at physician’s choice with 2:1 randomization. A total of 302 patients were randomized, 205 being assigned to receive olaparib and 97 to receive standard therapy. Most of patients received previous chemotherapy for MBC (71%). Median PFS was significantly longer in the olaparib group than in the standard therapy group (7.0 months vs. 4.2 months; HR 0.58, 95% CI: 0.43, 0.80; *p* ≤ 0.001). No significant difference in overall survival was found between olaparib and standard therapy, but data were still immature. Fewer patients in the olaparib arm experienced grade ≥3 adverse events compared to the standard therapy arm. Quality of life data (based on Global HRQoL) were significantly better in the olaparib group.

Based on these results, olaparib was the first PARPi to receive FDA approval for the treatment of patients with deleterious or suspected deleterious germline *BRCA1/2* mutation, HER2-negative metastatic breast cancer who have been presioulsy treated with chemotherapy either in the neoadjuvant, adjuvant, or metastatic setting.

Olaparib has also been evaluated in combination with platinum chemotherapy. Balmana et al. reported the combination of olaparib continuously to cisplatin 75 mg/m^2^ on day 1 of each 21-day treatment cycle in 59 patients with multiple metastatic cancers including 42 breast cancers [68]. This combination was tolerable only after adjusting the doses of olaparib and cisplatin (olaparib 50mg bid, days 1–5 associated to cisplatin 60 mg/m^2^). In patients with breast cancer with known *BRCA 1/2* mutation, ORR was 71% (12/17 assessable patients). In the same indication, Lee et al. evaluated the combination of olaparib to carboplatin [69]. Olaparib capsules 400 mg every 12 hours on days 1 to 7 with carboplatin AUC5 was a safe association. ORR was 87.5% in the *BRCA1* breast cancer cohort.

### 3.3. Talazoparib

In 2017, De Bono et al. [70] conducted the first-in-human dose escalation trial of talazoparib in patients with advanced solid tumors harboring germline *BRCA1/2* mutation or tumours potentially sensitive to PARP inhibitors. PARP inhibition was observed at doses ≥0.60 mg/day. The most common toxicity was hematologic with transient and reversible cytopenias easily managed with drug interruption and/or dose reduction. This trial included 14 breast cancer (all with deleterious BRCA1/2 mutations) treated with talazoparib at 1 mg/kg. The objective response rate (ORR) was 50% with one complete response.

When compared in vitro to olaparib and rucaparib, talazoparib has the highest trapping activity of PARP-DNA complexes at sites of single-strand DNA breaks [61]. Turner et al. reported a phase II trial that assessed talazoparib in patients with advanced breast cancer associated with germline *BRCA* mutation previously exposed to platinum agents (cohort 1) or to multiple prior cytotoxic regimens without previous exposure to platinum agents (cohort 2) [71]. The ORR was 21% (95% IC 10, 35) in cohort 1 and 37% (95% IC 18, 39) in cohort 2. Talazoparib had a manageable safety profile. The most common adverse event and reason for dose reduction was anaemia.

*EMBRACA* was the first open-label phase III trial by Litton et al that compared talazoparib to chemotherapy in patients with advanced breast cancer and germline mutations in *BRCA1* and *BRCA2* [72]. The patients had not received more than three prior cytotoxic regimens for advanced breast cancer. One-third of population were in the front line treament. The study randomized (2:1) 431 patients to receive talazoparib (1.0 mg) once daily or physician’s choice of standard single-agent chemotherapy (capecitabine, eribulin, gemcitabine or vinorelbine). Median PFS was 8.6 months (95% CI: 7.2–9.3) for patients treated with talazoparib and 5.6 months (95% CI: 4.2, 6.7) for those treated with chemotherapy (HR: 0.54 (95% CI: 0.41–0.71), *p* < 0.0001). ORR in the talazoparib group was more than twice that of the control arm (62.6% for talazoparib vs. 27.2% for chemotherapy (OR: 4.99 (95% CI: 2.9–8.8), *p* < 0.0001)). This clinical benefit was consistent in most subgroups, including hormone receptor status (TNBC or hormone receptor-positive), history of central nervous system metastases, *BRCA* mutation (*BRCA1* or *BRCA2*) or according to previous exposure to chemotherapy. Of note, in the small-sized subgroup of patients previously exposed to platinum-based chemotherapy, the benefit was uncertain. Safety profile with talazoparib was consistent with findings from previous trials. Hematologic grade 3 or 4 adverse events occurred in 55% of the patients who received talazoparib (especially anemia) and in 38% of the patients who received chemotherapy. This toxicity was not associated with clinical sequelae, and did not result in drug discontinuation. Patients treated with talazoparib experienced a significant increase in their global health status and quality of life compared with baseline, as opposed to chemotherapy-treated patients (3.0 (95% CI 1.2, 4.8) versus 5.4 (95% CI -8.8, -2.0); *p* < 0.0001) [80]. Recently, a meta-analysis pooled the results of the Olympiad and EMBRACA studies. This meta-analysis confirms that the use of a PARPi instead of a single chemotherapy can significantly delay the deterioration of the quality of life (QoL) (HR 0.40 (95% CI 0.29 to 0, 54)) [81]. The FDA has approved talazoparib for treatment of metastatic breast cancer patients carrying *BRCA1/2* germline mutation.

### 3.4. Veliparib

Veliparib has been essentially developed in breast cancer in concomitant association with platin-based chemotherapy. The *BROCADE* trial was a randomized phase 2 study that evaluated the association of carboplatin to taxane (paclitaxel) and PARP inhibitor (veliparib) in patients with *BRCA 1/2*-associated locally recurrent or metastatic breast cancer. A third arm evaluated the combination of veliparib with temozolomide, an alkylating agent, which was found to be highly synergistic with PARPi in preclinical and early clinical studies. The response rate in patients treated exclusively with carboplatin and paclitaxel was 61.3% that supported the data emerging from the *TNT* trial favoring the use of platinum compounds in *BRCA1/2*-associated breast cancer [44]. In the veliparib plus temozolomide arm, results were disappointing with median PFS (7.4 months (95% CI, 5.9–8.5)), median OS (19.1 months (14.3–21.3)), and ORR (28.6%, 20/70) being inferior to carboplatin-paclitaxel. Yet, the addition of veliparib to carboplatin/paclitaxel significantly improved response rate (ORR from 61.3% to 77.8 % (*p* = 0.027)), whereas PFS did not differ significantly. A phase III trial is currently ongoing evaluating these two platin-based schedules.

*I-SPY 2* is a multicenter phase II trial using adaptive randomization as a platform for screening multiple experimental regimens, combined with standard neoadjuvant chemotherapy for the treatment of high-risk stage II/III breast cancer. Tumors were classified into eight subtypes of biomarkers based on HER2 expression, hormone receptor expression, and 70-gene assay. Rugo et al. reported the results of the PARPi (veliparib)–carboplatin combination that was considered for HER2-negative tumors [73]. Patients were randomized to combined veliparib-carboplatin and standard chemotherapy (paclitaxel, followed by doxorubicin plus cyclophosphamide) or standard chemotherapy alone. A total of 72 patients were randomly assigned to receive veliparib–carboplatin including 17% with a mutation that was deleterious or suspected to be deleterious in *BRCA1* or *BRCA2*. Pathological complete rates in the TNBC population were 51% (95% Bayesian probability interval (PI), 36 to 66%) in the veliparib–carboplatin group versus 26% (95% PI, 9 to 43%) in the control group. It was not possible to evaluate individually the contribution of the PARP inhibitor and carboplatin.

In the same population, *BrighTNess* was a phase III trial designed to evaluate the addition of carboplatin with and without veliparib to the standard neoadjuvant combination of paclitaxel followed by doxorubicin and cyclophosphamide in 634 TNBC patients [74]. The results confirmed that adding carboplatin to standard chemotherapy increases the pCR rate (53 and 58% in the two arms containing carboplatin vs. 31% without carboplatin). However, the addition of veliparib did not increase pCR. In addition, 47 (51%) of 92 patients with a germline *BRCA* mutation achieved a pCR versus 262 (48%) of 542 patients without germline *BRCA* mutation, suggesting rather counterintuitively that the benefit of platinum was not driven by patients carrying *BRCA1/2* germline mutations.

### 3.5. Other PARPi

Drew et al. evaluated the anti-tumor activity of rucaparib, in a phase II, multicenter trial including germline *BRCA* mutation carriers with advanced breast cancer and ovarians cancer [76]. Intravenous and subsequently oral rucaparib were assessed. In the IV cohort, 44% (8/18) with germline *BRCA*-associated breast cancer experienced stable disease for 12 weeks. In the oral cohort, 20% (1/5) experienced stable disease for 12 weeks. There was no partial response or complete response. Rucaparib was well tolerated in patients up to doses of 480 mg per day. Rucaparib has also been evaluated in the treatment of metastatic breast cancer in combination with standard chemotherapy. Wilson et al. assessed escalading doses of intravenous rucaparib combined with carboplatin, carboplatin/paclitaxel, cisplatin/pemetrexed, or epirubicin/cyclophosphamide in a population of multiple advanced solid tumors including 22 breast cancers (seven with germline *BRCA* mutations) [77]. This phase I study showed a clinical activity even in heavily pretreated patient (one PR and one CR out of the germline *BRCA*-mutated breast cancers). Neutropenia and thrombocytopenia were the most common grade ≥3 toxicities.

The phase II study of the of Hoosier Oncology Group BRE09-146 assessed rucaparib in association with cisplatin for the treatment of TNBC or known *gBRCAm* patients with residual disease after anthracycline and/or taxane therapy [78]. 128 patients were assigned 1:1 to cisplatin alone or combination of cisplatin/rucaparib, followed by rucaparib single-agent. The addition of Rucaparib did not improve the 2-year disease free progression (63.1% vs. 58.3%, *p* = 0.43). Toxicity was similar in both arms.

Sandhu et al assessed niraparib in germline *BRCA* mutation carriers and patient with sporadic cancer. The maximum tolerated dose was reached at 300 mg/day. Out of the 22 patients with metastatic breast cancer, two patients achieved PR in the germline *BRCA*-mutated population [79]. Toxicity was manageable with essentially cytopenias, fatigue and digestive adverse events. A phase III (*BRAVO* trial) is comparing niraparib with chemotherapy of physician’s choice for patients with HER2-negative, germline *BRCA*-mutated breast cancer, similar to the *OlympiaD* and *EMBRACA* trials.

### 3.6. Mechanims of Resistance to PARPi

A number of potential mechanisms of resistance to PARP inhibitors have been suggested in preclinical models and confirmed in some clinical case reports, even though their actual clinical prevalence remains to be established. Thus, in a *BRCA2*-mutated pancreatic cancer cell line model which was rendered resistant to a PARP inhibitor, intragenic deletions of the initial frameshift mutation were identified, leading to restoration of the open reading frame. Such acquired mutations were shown to be associated with restoration of a competent HR repair in vitro and were also identified in tumor tissue from carboplatin-resistant ovarian cancer patients carrying the same baseline mutation [82]. Similar revertant mutations were also found in tumor materials from breast and ovarian cancers that had become resistant to olaparib, after an initial response [83]. These acquired mutations could be promoted via the error-prone alternative DNA repair pathways that are activated upon PARP inhibition in the context of HR deficiency [82].

Restoration of a functional HR repair under PARP inhibitors may also be obtained via *BRCA*-independent mechanisms. Thus, the loss of proteins favoring DSBs repair via NHE such as 53BP1 or REV7 was identified in different models of olaparib-resistant *BRCA1* cancer cell lines and was shown in vitro to promote a competent HR repair [84,85]. Other mechanisms of resistance to PARP inhibitors may include the loss of various proteins regulating the stability of the replication fork such as CDH4, PTIP, MLL3/4 and PARP1 itself, as well as overexpression of pump-efflux proteins which lead to reduced intra-cellular drug concentration, such as P-glycoprotein [84,86,87]. Of note, many of the above described mechanisms are acquired during treatment but little is known about primary resistance, which is a highly relevant clinical issue, since nearly 40% of patients with *BRCA1/2*-associated breast cancer patient have no detectable response when receiving PARP inhibitors.

### 3.7. Innovative Association with PARPi

#### 3.7.1. Association with Chemotherapy

A large number of therapeutics, including various chemotherapeutic agents or radiation therapy, have the potential to be synergistic with PARP inhibition and are currently under investigation [88]. Here, the patient population is not necessarily carrying *BRCA1/2* germline mutation or even BRCAness phenotype, since it is hypothesized that PARP inhibition would potentiate the DNA lesions induced by other treatments. However, even though promising results were obtained at preclinical level, the concomitant association of PARP inhibition with cytotoxic chemotherapy, including temozolomide, platinum compounds, gemcitabine or topo-isomerase inhibitors has been difficult to implement in the clinic due to potential additive hematological toxicities, resulting in infra-optimal doses of chemotherapy and/or PARP inhibitors and mitigated clinical results to date [68,69,77,89]. In the most advanced stage of development in breast cancer and as already mentioned earlier, the combination of veliparib with carboplatin-paclitaxel or temozolomide regimen in *BRCA1/2*-associated advanced breast cancer was demonstrated to be feasible but efficacy was uncertain [75].

#### 3.7.2. Association with radiation therapy

Another attractive therapy to combine with PARP inhibitors is radiation therapy, since it induces DNA damages, which are thought to rapidly recruit and activate PARP for DNA repair. Again, preclinical studies have shown synergistic effects and preliminary results of clinical trials have been promising. Thus, radiotherapy was tested in concomitant association with veliparib in brain metastases from breast or lung cancer and appeared to be well tolerated with preliminary signs of anti-tumor efficacy [90].

#### 3.7.3. Association with Other Targeted Therapies

Based on promising preclinical data, PARP inhibitors could also be associated with targeting of several oncogenic pathways such as EGFR, IGF, VEGF or PI3K. The latter axis was shown to be involved in the detection of DNA lesions and the regulation of BRCA1/2 expression. It has been suggested that suppressing PI3K/mTOR activity may result in generating BRCAness phenotype regardless of actual BRCA1/2 genotype. Consequently, clinical trials evaluating combination of PI3K or mTOR inhibitors with PARP inhibition have been initiated, with some positive preliminary positive results in early phase studies [91]. In addition, combination with drugs targeting cell cycle checkpoint regulators such as WEE1, ATR or CHK1 (which are involved in limiting entry in cell cycle, and thus allow optimal repair of DNA lesions induced by PARP inhibitors) may also be synergistic and are currently tested in the clinic [92].

#### 3.7.4. Association with Immune Checkpoint Inhibitors

Finally, PARP inhibition could be advantageously associated with emerging immune checkpoint inhibitors in *BRCA1/2*-mutated breast cancers, based on several observations. First, *BRCA*-associated breast cancer, notably the *BRCA1*-mutated forms were shown to display a higher immune infiltrate and a higher tumor mutational burden, both being associated with a higher probability of benefit from immunotherapy [93]. Second, olaparib exposure was shown to increase PD-L1 expression in BRCA1/2-mutated breast cancer cell lines or xenograft, which was associated with inhibition of T lymphocyte cytotoxicity, whereas combining olaparib with a monoclonal anti-PD1 revealed to be synergistic. Accordingly, clinical studies are ongoing testing such combinations in advanced breast cancer patients with encouraging preliminary results. The MEDIOLA study evaluated olaparib in combination with durvalumab, an anti-PD-L1 monoclonal antibody, in HER2-negative, *BRCA1/2*-associated metastatic breast cancer and reported a promising disease control rate of 80% at 12 weeks in the first 25 patients analyzed, independently from hormone receptor expression or BRCA type of mutation [94]. Similarly, in the TOPACIO study pembrolizumab (anti-PD1 monoclonal antibody) and niraparib were administered in metastatic TNBC patients and 28% of the 46 evaluable patients had objective response, the response rate being higher in patients with mutations in *BRCA1/2* or HR genes found in the tumor tissue [95].

## 4. Extending the Patient Population Candidate to DNA-Damaging Agents: The *BRCA*ness Concept

If biomarkers associated with a lack of sensitivity to PARP inhibitors or platinum compounds may help better selecting which patients with *BRCA1/2* germline mutation have the lowest probability of benefit, another issue is the identification of patients, which may have sensitivity to these classes of drugs, in spite of carrying *BRCA1/2* wild-type genes at the constitutional level. This is the “*BRCA*ness” phenotype, in which molecular alterations in the tumor drive a defective HR repair process, which may further broaden the patient population potentially candidate to DNA-damaging approaches. Yet, this putative patient population and its molecular features remain to be clearly identified. First, the expression of *BRCA1/2* genes in the tumor may be repressed by hypermethylation of gene promoter [96,97,98], or *BRCA1/2* genes may display somatic mutations [99], even though the impact of these alterations on PARP inhibitors and/or platinum sensitivity remains elusive. In addition, a number of individual genes known to be involved in HR repair, such as *ataxia telangiectasia mutated* (*ATM*), *ataxia telangiectasia and Rad3-related* (*ATR*), *CHEK1*, *CHEK2*, *deleted in split hand/split foot protein 1* (*DSS1* or *SHFM1*), *RAD51*, *Nijmegen breakage syndrome protein 1* (*NBS1* or *NBN*) and the *Fanconi anaemia complementation group* (*FANC*), *cyclin-dependent kinase 12* (*CDK12*) and *excision repair cross-complemntation group 1* (*ERCC1*), *phosphatase and tensin homolog* (*PTEN*) and *BRCA1-interacting protein 1* (*BRIP1*) genes, may display alterations (either germline or somatic) that are associated with sensitivity to DNA-damaging agents [100]. Comprehensive analyses of these genes have been performed in ovarian cancer and have shown that up to 50% of patients may have a BRCAness phenotype [97,101], which correlates with sensitivity to platinum compounds [102]. A similar phenotype was identified in breast cancer patients, with a significant number of *BRCA1/2* somatic mutations (up to 20%) and somatic or germline mutations in genes involved in HR repair [99], especially in the TNBC subtype [103,104]. In addition, transcriptional signatures have been generated that distinguish *BRCA1/2*-associated and sporadic ovarian or breast cancer and may be able to recognize tumors with HR deficiency, *BRCA*ness phenotype and/or PARP sensitivity [105,106,107,108].

Another way of identifying *BRCA*ness phenotype has been based on large scale genomic analysis and detection of specific mutational signatures associated with HR deficiency. These signatures are defined by the number and the type of mutation which can be observed in these tumors, in relation with the predominant error-prone mechanisms of DNA DSBs repair, as well as the type of structural rearrangements of the genome which can be generated in this context [56]. These “genomic scars” of HR deficiency can be detected by array-based comparative genomic hybridization, single-nucleotide polymorphism genotyping and massive sequencing technologies and may include loss of heterozygosity (LOH), telomeric allelic imbalance (TAI), and large-scale state transitions (LST), each element taken alone or being combined in a predictive score, as developed by various commercial companies [109,110,111]. All these structural rearrangement signatures have been shown to possibly correlate to DNA-damaging agents including PARP inhibitors, platinum compounds and/or alkylating agents [112,113,114,115]. Yet, in breast cancer, definitive evidences are still lacking that *BRCA*ness-associated breast cancer may respond to PARP inhibitors and a specific clinical trial is ongoing in France, testing whether rucaparib might be effective in advanced breast cancer without *BRCA1/2* germline mutation but either LOH or somatic *BRCA1/2* mutation as determined on metastatic tissue (Ruby trial, NCT02505048). Indeed, the predictive value of these tests may be dependent on the type of sample analyzed. For example, in the above-mentioned TNT trial, a HR deficiency score estimated by the Myriad Genetics test (MyChoice, based on combination of LOH, TAI and LST scores), was determined on the primary tumor and was not able to predict platinum benefit in advanced disease, possibly due to inability to capture the plasticity of *BRCA*ness phenotype during the disease course [44]. Various other *BRCA*ness-associated mutational signatures are under evaluation, including a more comprehensive, whole-genome sequencing derived, HR deficiency test (HRDetect), which was shown to identify up to 20% of breast cancer patients displaying such a phenotype [116]. In addition, estimating the tumor mutational burden, which is supposed to be higher in *BRCA1/2* deficient cancers, might be another method to identify *BRCA*ness [117].

Finally, functional tests evaluating the ability of tumor cells to activate the HR process may also be used to define which cancers have the highest probability of response to DNA-damaging agents that mobilize this mechanism of DNA repair. For example, the recruitment of RAD51 at DNA DSBs, as identified by RAD51 foci by IHC, was recently found to be associated with PARP inhibitors resistance in preclinical and clinical models and might be a robust biomarkers of HR deficiency, which may complement or substitute the above-described genomic-based approaches [118]. 

## 5. Conclusions

While being initially essentially confined to the field of screening and/or prevention, the knowledge of a *BRCA1/2* germline mutation has now become a major biomarker for therapeutic decision in advanced breast cancer management. In HER2-negative disease, these patients may clearly benefit from specific therapeutic targeting, including platinum compounds and PARP inhibitors, and clinical investigations are underway to evaluate their impact in earlier settings. Yet, their optimal use will need additional works to understand and overcome mechanisms of resistance and to better define which patients might most benefit, including the likely larger than previously anticipated, but still to precisely identify, *BRCA*ness population. In addition, various attractive combinations associating PARP inhibitors with other therapeutic strategies, including radiation therapy, cytotoxic chemotherapies, targeted therapies and recently emerging immunotherapies, have the potential to further increase their clinical benefit. Finally, the increasing deciphering of the complexity of DNA-damage response process and its deregulation in cancer may further broaden the therapeutic resources exploiting this pathway, ultimately leading to major clinical advances in breast cancer patients.

## Figures and Tables

**Table 1:** *BRCA 1/2*-associated Breast cancer and Platinum agents.

Trial	Phase	Patients	Dose and Schedule	Response
**Neoadjuvant Setting**				
Byrsky 2010 [29]	Retrospective	*gBRCA1m* (*n* = 102) including 12 pts treated with cisplatin	-	pCR = 83% (10/12)
Byrsky 2011 [38]	Retrospective	*gBRCA1m* (*n* = 28)	Cisplatin 75 mg/m^2^ every 3 weeks 4 cycles	pCR = 60.5% (23/28)
Byrsky 2014 [39]	Retrospective	*gBRCA1m* (*n* = 107)	Cisplatin 75 mg/m^2^ every 3 weeks 4 cycles	pCR = 61% (65/107)
Silver 2010 [40]	Retrospective	TNBC (*n* = 28) including 2 pts with *gBRCA-1m*	Cisplatin 75 mg/m^2^ every 3 weeks 4 cycles	pCR = 22% (6/28) including 100% (2/2) in pts with *gBRCA-1m*
Hahnen 2017 [41]	Phase II	TNBC (*n* = 146) including 50 pts with *gBRCAm*	Carboplatin AUC 1.5 or AUC 2 every week 18 weeks following standard CT	pCR = 56.8% (83/146)
**Metastatic Setting**				
Byrsky 2012 [42]	Phase II	*gBRCA1m* (*n* = 20)	Cisplatin 75 mg/m^2^ every 3 weeks 6 cycles	ORR = 80% (9 CR; 7 PR) mPFS = 12 month
Isakoff 2015 [43]	Phase II	TNBC or BRCA associated BC (*n* = 83) including 11 pts with *gBRCAm*	Cisplatin 75 mg/m^2^ or Carboplatin AUC6 every 3 weeks	ORR = 54.5% in pts with *gBRCA1/2-m*
Tutt 2018 [44]	Phase III	TNBC or BRCA associated BC (*n* = 376) including 43 pts with *gBRCAm*	Carboplatin AUC 6 vs. Docetaxel 100 mg/m^2^ every 3 weeks 6 cycles	ORR = 68%vs 33.3% in pts with *gBRCAm,* PFS = 6.8 mo vs. 4.8 (*p* = 0.002) in pts with *gBRCAm*

*gBRCAm* = germline BRCA mutation, *TNBC* = triple negative breast cancer, *pts* = patients, *pCR* = pathologic complete response, *ORR* = overall response rate, *PFS*: progression-free survival, *CT* = chemotherapy.

**Table 2:** *BRCA1/2*-associated breast cancer and PARP inhibitors.

Trial	Phase	Patients	Dose and Schedule	Response in Breast Cancer
**Olaparib**				
Fong 2009 [63]	I	Solid advanced tumors (*n* = 60) including 9 MBC with 3 *gBRCAm* carriers	Olaparib 10 mg to 600 mg BID 2/3 weeks	1 CR; 1 SD; 1 PR
Tutt 2010 [64]	II	*gBRCAm* associated BC (*n* = 27)	Olaparib 400 mg BID (first cohort) Olaparib 100 mg BID (second cohort)	ORR = 41% (first cohort) ORR = 22% (second cohort)
Gelmon 2011 [65]	II	Solid advanced cancer (*n* = 91) including 26 advanced TNBC; 10 with *gBRCAm*	Olaparib 400 mg BID	PFS = 3.6 mo vs. 1.8
Kaufman 2015 [66]	II	*gBRCAm* associated tumors (*n* = 298) including 62 BC	Olaparib 400 mg BID	ORR = 12.6%
Robson 2017 [67]	III	*gBRCAm* associated BC (*n* = 302) olaparib (*n* = 205) vs. SOC (*n* = 97)	Olaparib 300 mg BID vs. SOC	ORR = 59.9% vs. 28.8% PFS = 7.0 mo vs. 4.2
**Olaparib + CT**				
Balmana 2014 [68]	I	Solid advanced tumors (*n* = 59) including 42 BC	Olaparib 50–200 mg BID Cisplatin 75 mg/m^2^	ORR = 71% (*gBCRA* population)
Lee 2014 [69]	I/Ib	Solid advanced tumors (*n* = 45) including 8 BC	Olaparib 100–400 mg BID Carboplatin AUC5	ORR = 87.5% (*gBRCA1* population)
**Talazoparib**				
De Bono 2017 [70]	I	Solid advanced tumors (*n* = 110) including 14 BC treated with talazoparib	Talazoparib 0.025 mg to 1 mg daily	ORR = 50% with 1 CR
Turner 2017 [71]	II	*gBRCAm* associated BC (*n* = 84) cohort 1: 49 pts cohort 2: 35 pts	Talazoparib 1 mg daily	ORR = 21% (cohort 1) ORR = 37% (cohort 2)
Litton 2018 (EMBRACA) [72]	III	*gBRCAm* associated BC (*n* = 431) talazoparib (*n* = 247) vs. SOC (*n* = 144)	Talazoparib 1 mg daily vs. SOC	ORR = 62.6% vs. 27.2% PFS = 8.6 mo vs. 5.6
**Veliparib + CT**				
Rugo 2016 (I SPY) [73]	II	Stage II/III BC 72 pts assigned to veliparib-carboplatin including 17 *gBRCAm*	Veliparib 50 mg BID associated to carboplatin vs. SOC	pCR = 51% vs. 26%
Loibl 2016 (Brightness) [74]	II	Stage II/III BC 634 pts with TNBC including 92 *gBRCAm*	Veliparib 50 mg BID or placebo associated to SOC	pCR = 51% in *gBRCAm* treated with veliparib
Han 2018 [75]	II	*gBRCAm* associated BC (*n* = 284)	Veliparib 120 mg daily or placebo associated with carboplatin/paclitaxel or temozolomide	Veliparib/temozolomide ORR = 26%; PFS = 7.4 mo
				Veliparib/carboplatin/taxol ORR = 77.8% vs. 61.3% PFS = 14.1 mo vs. 12.3
**Rucaparib**				
Drew 2016 [76]	II	Solid advanced tumors (*n* = 78) including 27 BC	Rucaparib IV and PO 92 mg to 600 BID	SD = 44% (8/18 BC) in the IV cohort SD = 20% (1/5 BC) in the PO cohort
**Rucaparib + CT**				
Wilson 2017 [77]	I	Solid advanced tumors (*n* = 85) including 22 BC with 7 *gBRCAm* carriers	Rucaparib IV 12–24 mg then PO 80–360 mg Chemotherapy	1 CR and 1 PR in *gBRCAm* population
Miller 2015 [78]	II	Residual tumor post neoadjuvant CT (*n* = 128) *gBRCAm* or TNBC	Rucaparib 25–30 mg IV days 1 to 3 (4 cycles) then rucaparib PO 100 mg weekly Cisplatin 75 mg/m^2^	2 yr DFS = 58.3% (cisplatin alone) vs. 63.1% (cisplatin/rucaparib)
**Niraparib**				
Sandhu 2013 [79]	I	Solid advanced tumors (*n* = 100) including 22 BC	Niraparib 30–400mg daily	2 PR in the *gBRCAm* population

*gBRCAm* = germline BRCA mutation, pts = patients, mo = months, MBC = metastatic breast cancer, TNBC = triple negative breast BC, ORR = overall response rate, PFS: progression-free survival, DFS = Disease-free survival, SOC = standard of care, SD = stable disease, PR = partial response, CR = complete response, IV = intra-venous, PO = per os, CT = chemotherapy.
